# Early alterations in vascular contractility associated to changes in fatty acid composition and oxidative stress markers in perivascular adipose tissue

**DOI:** 10.1186/1475-2840-9-65

**Published:** 2010-10-21

**Authors:** Alejandro Rebolledo, Oscar R Rebolledo, Carlos A Marra, María E García, Ana R Roldán Palomo, Laura Rimorini, Juan J Gagliardino

**Affiliations:** 1 GINFIV - Grupo de Investigación en Fisiología Vascular, Grupo Vinculado a CENEXA (UNLP - CONICET LA PLATA), Facultad de Ciencias Exactas, Universidad Nacional de La Plata, La Plata, Argentina; 2 CENEXA - Centro de Endocrinología Experimental y Aplicada, UNLP-CONICET LA PLATA, Centro Colaborador OPS/OMS), Facultad de Ciencias Médicas, Universidad Nacional de La Plata. La Plata, Argentina; 3 INIBIOLP - Instituto de Investigaciones Bioquímicas de La Plata, UNLP-CONICET LA PLATA, Cátedra de Bioquímica y Biología Molecular, Facultad de Ciencias Médicas, Universidad Nacional de La Plata, La Plata, Argentina

## Abstract

**Aim:**

To test the early effect of fructose-induced changes in fatty acid composition and oxidative stress markers in perivascular adipose tissue (PVAT) upon vascular contractility.

**Methods:**

Adult male Wistar rats were fed a commercial diet without (CD) or with 10% fructose (FRD) in the drinking water for 3 weeks. We measured plasma metabolic parameters, lipid composition and oxidative stress markers in aortic PVAT. Vascular contractility was measured in aortic rings sequentially, stimulated with serotonin (5-HT) and high K^+^-induced depolarization using intact and thereafter PVAT-deprived rings.

**Results:**

Comparable body weights were recorded in both groups. FRD rats had increased plasma triglyceride and fructosamine levels. Their PVAT had an increased saturated to mono- or poly-unsaturated fatty acid ratio, a significant decrease in total superoxide dismutase and glutathione peroxidase activities and in the total content of glutathione. Conversely, lipid peroxidation (TBARS), nitric oxide content, and gluthathione reductase activity were significantly higher, indicating an increase in oxidative stress. In aortic rings, removal of PVAT increased serotonin-induced contractions, but the effect was significantly lower in rings from FRD rats. This effect was no longer observed when the two contractions were performed in PVAT-deprived rings. PVAT did not affect the contractions triggered by high K^+^-induced depolarization either in CD or FRD rats.

**Conclusions:**

FRD induces multiple metabolic and endocrine systemic alterations which also alter PVAT and the vascular relaxant properties of this tissue. The changes in PVAT would affect its paracrine modulation of vascular function.

## Background

The metabolic syndrome (MS) is characterized by central adiposity, insulin resistance (IR), impaired glucose tolerance (IGT) or type 2 diabetes, hypertension and dyslipidemia. These factors predispose to greater risk for developing cardiovascular disease [1]. The increased consumption of unhealthy diets - particularly in Western societies - and a sedentary behavior have greatly contributed to the high prevalence of this condition [2]. In this context, fructose consumption has largely increased as a result of its addition to soft drinks and processed food [3].

Sucrose- or fructose-rich diets administered to normal rats induce metabolic alterations that resemble the profile of the human MS [4-8]; consequently, the fructose-rich diet (FRD) constitutes a useful model for studying the role of unbalanced diets in the pathogenesis of the MS and cardiovascular disease. It has been shown that FRD promotes decreases in endothelial NO production [9] and vasorelaxation [10,11] as well as impaired insulin-induced vasodilation [9]. Further, the FRD model is also useful to test the effects of different preventive strategies aimed at decreasing MS vascular morbidity and mortality.

We have recently shown that rats fed a FRD present marked changes in plasma lipid and hormone profile, oxidative damage to lipoprotein fractions and their fatty acids (FAs), fat content in the liver, and fatty acyl composition of total lipids in abdominal adipose tissue. These multiple abnormalities were associated with significant changes in insulin sensitivity and IGT, marked changes in liver glucose metabolism, decreased pancreatic β-cell mass, and increased production of reactive oxygen species (ROS) [12-16]. The latter effect, commonly described as oxidative stress, contributes to the development and progression of diabetes micro and macrovascular complications [17-19]. Supporting our data, it has been reported that FRD promotes oxidative damage, reducing antioxidant defenses and enhancing ROS production [3,12,20-22]. Time-sequential studies have detected enhanced ROS production as early as one week after FRD administration in aorta, heart and polymorphonuclear cells; besides, vascular and cardiac hypertrophy together with a rise in blood pressure have also been observed [23]. It is not known, however, whether these multiple and early FRD-induced disturbances also affect perivascular adipose tissue (PVAT).

The PVAT consists of a thin layer of fat located outside the adventitial layer, surrounding most systemic blood vessels. Originally, it was considered as a simple mechanical support for vasculature; however, recent studies have demonstrated that PVAT plays an active modulatory role on vessel function in a paracrine fashion through the release of either contractile or relaxant substances which act on the underlying vascular smooth muscle[24-29]. ROS are among the vasoactive products released by PVAT [30,31]. Additionally, recent studies performed *in vivo *have demonstrated a relationship between PVAT and insulin resistance: insulin sensitivity was negatively correlated with the amount of PVAT at the brachial artery [32].

In an attempt to provide an insight into the relationship of impaired PVAT metabolism/function and vascular reactivity, we fed normal rats with a FRD for 3 weeks to test: a) whether FRD reproduces the changes observed in abdominal adipose tissue (fatty acyl compositional changes and increased markers of oxidative stress) in PVAT, and b) the potential effect of these PVAT changes upon the vascular reactivity of aorta rings from control and FRD rats in response to different stimuli.

## Methods

### Materials

Thiobarbituric acid, CoA, N-ethylmaleimide, NAD+, NADH(H), NADPH(H), standards for oxidized and reduced glutathione, serotonin (5-HT), organic and inorganic components for buffer preparations, teraetoxypropane and butylated hydroxytoluene were purchased from Sigma Chemical Co (St. Louis, Mo, USA). Sodium nitrite (99.8% pure) was from Mallinckrodt (Griesheim, Germany). All solvents were HPLC-grade and provided by Carlo Erba (Milano, Italy). Lipid standards for c-gas liquid chromatography (c-GLC) identification were from Serdary Research Laboratories (London, Ontario, Canada). All standards were stored in appropriate solvents under nitrogen atmosphere at -20°C. Concentrations and purities were routinely checked by HPLC and/or c-GLC. Other chemicals used were reagent grade and obtained from local commercial sources.

### Animals, plasma determinations and characteristics of the FRD-induced insulin resistance model

Normal male Wistar rats weighing 180-200 g were maintained in a temperature-controlled environment (23°C) with a fixed 12-hour light/dark cycle and fed a standard laboratory rat diet (Ganave, Argentina) for one week (stabilization period); the compositional analysis of this diet has been described elsewhere [33]. Rats were fed according to the American Institute of Nutrition [34]. After that period, rats were divided into two groups and fed the above mentioned diet and tap water (control diet, CD) during 3 weeks, or the same diet with the addition of 10% fructose into the drinking water (FRD). The fructose solution was freshly prepared and changed daily. In both groups, the relative FA content in the diet was 1.5% *16:0*, 2.1% *18:0*, 26.6% *18:1*, 57.3% *18:2 n-6*, 1.4% *18:3 n-3*, 0.2% *20:3 n-6*, 0.2% *20:4 n-9*, 0.2% *20:1*, and other FAs in trace amounts (< 0.2%) sufficient to cover the animals' nutritional requirements. Before sacrifice, blood samples were obtained from the retroorbital plexus under isoflurane (Florane, Abbot, Argentine) anesthesia; the samples were placed into chilled tubes with heparin, rapidly centrifuged at 4°C at 300 × g, and immediately analyzed or stored at -20°C. Plasma triglycerides, glucose and fructosamine were measured using commercial kits (Bio Systems Diagnostics, Argentina) implemented in an automated clinical analyzer. Insulin levels were determined by radioimmunoassay using rat insulin standard. Insulin resistance was assessed with the HOMA score, calculated with the formula: fasting plasma insulin (μU/ml) × fasting plasma glucose (mmol/l)/22.5, as described by Matthews et al. [35].

All the experimental procedures followed during this investigation conformed to the Guide for the Care and Use of Laboratory Animals published by the US National Institutes of Health (NIH publication no. 85-23, revised 1996) and to the guidelines established by the Animal Welfare Committee of La Plata School of Medicine.

### PVAT measurements

After sacrifice, thoracic aortas were quickly removed and placed in Petri dishes with chilled PBS. The adhered PVAT was dissected under a stereoscopic zoom microscope (Model SMZ645/660, Nikon Instruments Inc., Melvilla, NY, USA). Aliquots of fat tissue weighing 60 to 80 mg were homogenized in a buffer solution containing 50 mM Tris-HCl (pH 7.4), 1.15% KCl, 1 mM N-ethylmaleimide and 0.1% BHT (v/v) with a glass-teflon microhomogeneizer. The homogenates were centrifuged at 4°C (600 × g) for 20 min and the resulting supernatants were assayed immediately or stored at -70°C under argon atmosphere. The extent of lipid peroxidation was determined by measuring thiobarbituric acid reactive species (TBARS) by the method of Yagi [36] and expressed as nmol TBARS/mg wet tissue. Teraetoxypropane (Sigma) was used as standard for calibration.

Glutathione and enzymatic antioxidant activity were also determined in PVAT. Total glutathione (GSSG + GSH) was measured following the recycling enzymatic assay described by Brigelius et al. [37]. Superoxide dismutase activities (SOD) were assayed as described by Flohé and Ötting [38]. Glutathione peroxidase (GSHPx) and glutathione reductase (GSHRd) were determined according to Habig et al. [39] and Carlberg and Mannervik [40], respectively.

Nitric oxide (NO) content in the homogenates was estimated as the sum of nitrates plus nitrites (NOx), which results as the spontaneous and stable end-point degradation of NO. NOx level was measured with the spectrophotometric method described by Miranda et al. [41] using sodium nitrite as standard.

### Lipid composition analysis

Appropriate aliquots of tissue homogenates were processed by c-GLC fatty acyl composition analysis. Briefly, total lipids were extracted by the method of Folch et al. [42]. c-GLC of fatty acid methyl esters (FAME) was performed as previously reported [43], except that in this case we used a capillary column (Supelco, Avondale, PA, USA) mounted on a Hewlett Packard HP 6890 Series GC System Plus (Avondale, PA) equipped with a terminal computer integrator system. FAME were identified by comparison of their relative retention times with authentic standards, and mass distribution was calculated electronically by quantification of the peak areas.

### Vascular reactivity studies

The aortas were cut into 3-4 mm wide rings and gently suspended between two stainless steel wires in a water-jacketed organ bath containing Krebs-Ringer bicarbonate (KRB) solution with the following composition (in mM): 130 NaCl, 4.7 KCl, 1.17 Na_2_HPO_4_, 1.16 MgSO_4_, 24.0 NaHCO_3_, 2.5 CaCl_2 _and 6 glucose. The solution was continuously bubbled with a mixture of 5% CO_2 _and 95% O_2_, pH 7.4, and kept at 37°C. The lower wire was fixed to a vertical plastic rod immersed in the organ bath, while the upper one was rigidly attached to a force transducer (Letica TRI-201). Initially, arterial rings were stretched until attaining a force of ≈ 2 g; thereafter, the rings were allowed to stabilize for 60 min and washed with fresh KRB every 20 min. The signals from the force transducers were amplified and driven into an analog-digital board (DT16EZ, Data Translation, Inc., Marlboro, MA, USA) mounted in a desktop computer. On-line recordings and files for later processing were obtained with the appropriate software (Labtech Notebook Pro, Laboratory Technology Corp., Wilmington, MA, USA).

In some experiments, a high-K^+ ^KRB solution with the following composition was used (in mM): 54.7 NaCl, 80 KCl, 1.17 Na_2_HPO_4_, 1.16 MgSO_4_, 24.0 NaCO3 H, 2.5 CaCl_2 _and 6.0 glucose.

To evaluate the potential modulatory role of adipose tissue, whereas some rings were dissected to remove the adherent PVAT, in others PVAT was left intact. In rings with an intact PVAT, a special protocol was developed to study the possible FRD-induced modifications on the relaxant effects of this tissue. For this purpose, a contraction was induced by 5 μM 5-HT, which acts through the activation of membrane receptors of vascular smooth muscle. Thereafter, the rings were carefully dismounted from the chambers and PVAT was carefully removed with micro scissors under a stereoscopic zoom microscope. Afterwards, the rings were remounted in the chambers as described before (including the 1-h stabilization), and the 5-HT challenge protocol was repeated. In order to minimize differences among vascular rings, the results of this second exposure to 5-HT were presented as paired to the first one, *i.e.*, the change in the maximal force of the second contraction was expressed as a percentage of the first one. To test the effect of a different vessel contraction stimulatory pathway, we used a similar protocol (challenge of the intact ring followed by the same stimulus applied to the same ring after PVAT removal), but muscle contraction was triggered by high K^+^-induced depolarization.

In order to study whether vascular smooth muscle was affected by the FRD, appropriate controls were performed removing PVAT from CD and FRD rings after isolation of the aorta; the denuded rings were then subjected to the same two sequential steps of the protocol described above, including the demounting and remounting step between the two contractions. Although initially studies were performed using either intact or fat-deprived aorta rings, we later adopted the above mentioned procedure to obtain most consistent and least dispersed data.

### Statistical analyses

Results are expressed as means ± SEM. One-way ANOVA followed by Bonferroni's test and Student's t-test were used for establishing statistically significant differences between groups. In all instances of statistical analysis, P < 0.05 was considered significant.

## Results

### Food intake, body weight and blood parameters

A comparable caloric intake was recorded in both experimental groups. CD and FRD rats consumed an average of 19.2 and 13.3 g/animal/day; FRD animals also consumed 5.26 g fructose (calculated from the amount of fructose solution drinked daily). Accordingly, the food composition for CD *vs. *FRD rats was 45% carbohydrates, 43% proteins and 12% lipids *vs. *60% carbohydrates, 31% proteins and 9% lipids. Consequently, there was a small but not significant difference in caloric intake between CD and FRD rats (55.65 ± 4.54 *vs. *50.79 ± 3.43 kcal/day, respectively), No differences were detected in initial and final body weight between the experimental groups.

There were no significant differences in fasting plasma glucose between groups (FRD *vs. *CD, 6.28 ± 0.15 *vs. *6.05 ± 0.15 mmol/L). FRD rats, however, had significantly higher plasma triglyceride, fructosamine and insulin levels (1.61 ± 0.14 *vs. *1.13 ± 0.08 mmol/L, P < 0.02; 162.6 ± 3.3 *vs. *146.2 ± 5.6 μmol/L, P < 0.03; 1.12 ± 0.04 *vs. *0.86 ± 0.10, P < 0.05) as well as HOMA insulin resistance index (7.66 ± 0.31 *vs. *5.16 ± 0.21, P < 0.001, respectively) than CD rats. These results validate the presence of an early fructose-induced metabolic disturbance and glucoxidative state in FRD rats, as described previously [12,13,15].

### PVAT: FA composition, antioxidant defense system, TBARS and NOx

The FA composition of total lipids extracted from PVAT of both experimental groups is shown in Table 1. FRD administration for 3 weeks increased the proportion of saturated myristic (14:0), palmitic (16:0) and stearic (18:0) acids by 37%, 17% and 24%, respectively. Conversely, the stearic acid derivative oleic acid (18:1) showed a significant 22% decrease. The polyunsaturated fatty acids (PUFAs) of the n-6 family showed an uneven behavior in FRD rats: the percentages of eicosatrienoic (20:3n-6) and arachidonic (20:4n-6) FAs decreased significantly (66% and 33%, respectively), while the linoleic (18:2n-6) and alpha and gamma-linolenic (18:3n-3) acid proportions remained unchanged. Thus, the total amount of PUFAs decreased significantly in FRD rats. Accordingly, the ratios of total saturated to mono-unsaturated FAs and total saturated FAs to PUFAs were higher in this experimental group.

**Table 1:** Fatty acid composition (mol %) of total lipids of PVAT from CD and FRD rats

FA	CD	FRD
**14:0 **(myristic acid)	1.9 ± 0.13	2.6 ± 0.10 ***
**16:0 **(palmitic acid)	27.6 ± 1.03	32.3 ± 0.70 **
**16:1 **(palmitoleic acid)	1.0 ± 0.09	0.5 ± 0.05 ***
**18:0 **(stearic acid)	19.4 ± 0.65	24.1 ± 0.65 ***
**18:1 **(oleic acid)	12.8 ± 0.35	10.0 ± 0.20 ***
**18:2 n-6 **(linoleic acid)	15.4 ± 0.33	15.9 ± 0.60
**18:3 n-3 **(α and γ linolenic acids)	0.2 ± 0.02	0.2 ± 0.01
**20:3 n-6 **(eicosatrienoic acid)	0.3 ± 0.02	0.1 ± 0.02 ***
**20:4 n-6 **(arachidonic acid)	21.6 ± 1.53	14.5 ± 0.90 ***
Σ **Sat**/Σ **Mono**	3.62 ± 0.08	5.72 ± 0.13 ***
Σ **PUFA**	37.5 ± 0.74	30.7 ± 0.45 ***
Σ **Sat**/Σ **PUFA**	1.31 ± 0.04	1.93 ± 0.03 ***

The values represent the means ± SEM; n = 11 animals for each group. Asterisks indicate P values for differences between CD and FRD; ***< 0.001, ** < 0.01.

CD: control; FRD: fructose-fed rats; Sat: saturated; Mono: monounsaturated; PUFA: polyunsaturated fatty acids.

FRD significantly modified those parameters related to the antioxidant defense system and the main biomarkers of oxidative damage (total GSH, TBARS and NOx). Total SOD and GSHPx activities as well as total glutathione content (GSH + GSSG) were significantly lower in PVAT homogenates of FRD rats. Conversely, TBARS content and total NO_x _and GSH-Rd activity were significantly higher in these rats (Table 2).

**Table 2:** Oxidative stress markers measured in PVAT of CD and FRD rats

	SOD (U/mg)	GSH-Px (U/mg)	GSH-Rd (U/mg)	TBARS (nmol/mg)	NO_x_ (pmol/mg)	GSH + GSSG (pmol/mg)
CD^a^	7.96 ± 0.45	12.14 ± 0.94	19.60 ± 1.10	0.27 ± 0.01	110.18 ± 5.89	88.40 ± 4.42
FRD^b^	4.98 ± 0.13	6.22 ± 0.27	26.85 ± 1.15	0.53 ± 0.03	215.38 ± 5.03	47.08 ± 1.98
a *vs. *b, P	< 0.001	< 0.001	< 0.001	< 0.001	< 0.001	< 0.001

Values represent the means ± SEM. n = 7 animals for each group. CD: control; FRD: fructose-fed rats; SOD: superoxide dismutase; GSH-Px: glutathione peroxidase; GSH-Rd: glutathione reductase; TBARS: thiobarbituric acid reactive species; NOx: nitrates plus nitrites; GSH+GSSG: total content of glutathione in PVAT.

### Vascular reactivity studies

**Figure 1:**
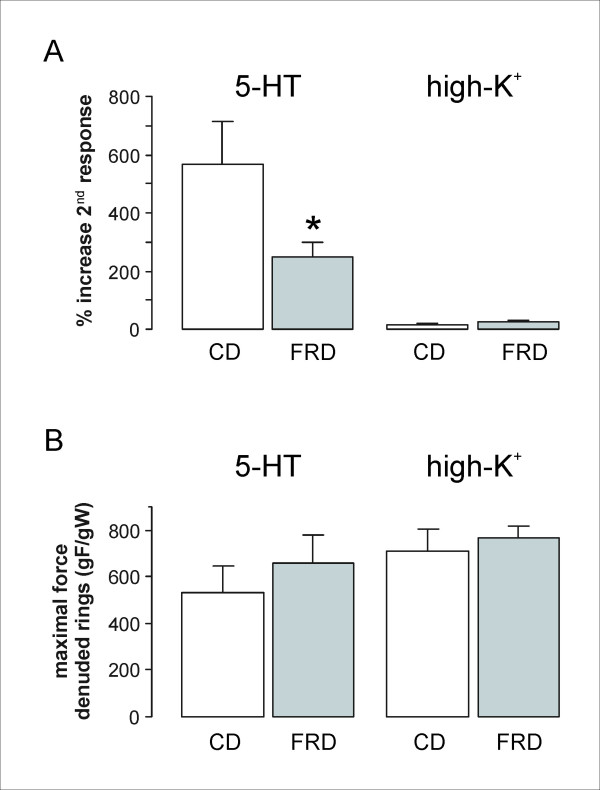
**Vascular reactivity experiments with and without perivascular adipose tissue in CD and FRD animals**. **A**. Increase in the maximal force of the second contraction (without PVAT) developed by aortic rings from CD and FRD animals stimulated with 5 μM 5-HT (CD, n = 17; FRD, n = 15) or high-K^+ ^KRB (CD, n = 20; FRD, n = 17). Results are expressed as the increase of the contraction without PVAT compared to a previous contraction with PVAT whose maximal force is taken as 100%. The asterisk indicates a statistically significant difference from the CD group. **B**. Maximal forces attained by denuded aortic rings (without PVAT) from CD and FRD animals stimulated with 5 μM 5-HT (CD, n = 8; FRD, n = 7) or high-K^+ ^KRB (CD, n = 8; FRD, n = 9).

Since it is known that PVAT attenuates the smooth muscle force development induced by 5-HT [24,25], we tested its effect (5 μM concentration) upon two paired contractions (intact and PVAT-deprived rings) of aorta rings obtained from CD and FRD rats (see above). Removal of PVAT increased the developed force; as shown in Figure 1A, the second contraction (ring without PVAT) was bigger than the first one (intact ring). In the case of aortic rings from FRD animals, however, the increased force recorded after PVAT removal was significantly smaller than the one observed in rings obtained from CD animals (Figure 1A). This effect was no longer observed when the two contractions were performed in PVAT-deprived rings (Figure 1B). Additionally, the second contractions in these rings showed only a small and comparable increase in either CD or FRD rats (data not shown).

A similar experimental protocol was performed using high extracellular K^+^, that triggers smooth muscle cell contraction by depolarization and subsequent opening of voltage-operated Ca^2+ ^channels. Results showed that PVAT does not affect the force development induced by depolarization; in fact, the percentage of increase of the second contraction (without PVAT) was small and of the same magnitude as that recorded in PVAT-deprived rings (Figure 1A). The lack of effect of PVAT on smooth muscle contractions induced by high extracellular K^+ ^was also observed in aortic rings obtained from FRD animals (Figure 1A). Furthermore, no differences were seen between CD and FRD PVAT-deprived rings in the maximal force produced by depolarization with high K^+ ^(Figure 1B).

## Discussion

### Blood parameters measured in the FRD- rats model

As already reported, we have currently recorded a significant increase in plasma fructosamine level in rats fed a FRD for 3 weeks, indicating an increment in systemic protein glycosylation [15,19]. Since no differences were recorded in fasting glucose levels but FRD rats had IGT, we must assume that these rats present abnormally high postprandial glucose levels. FRD rats also had higher circulating insulin levels and HOMA score values, thus supporting the presence of an IR state. Hypertriglyceridemia - another IR-inducer [3,12,15,22] - was also present in these rats due to a combined effect of excess FA provision to the liver, decreased esterification in adipose tissue, and attenuation of the lowering effect of insulin on hepatic VLDL secretion. Such changes can be ascribed to a) the increased hepatic *de novo *lipogenesis observed during high carbohydrate feeding [44], b) the inhibition of lipoproteinlipase (LPL) activity reported in FRD rats [45], and c) the consequent decrease in VLDL and chylomicron hydrolysis.

### PVAT measurements

#### Lipid composition analysis and PVAT redox state

In line with the above mentioned changes, PVAT fatty acyl lipid composition showed significant changes in FRD rats, mainly an increase in saturated FAs and a decrease in mono- and polyunsaturated FAs, resulting in a rise of the saturated/MUFA and saturated/PUFA ratios. We have previously reported similar changes in the abdominal adipose tissue of these rats, with increases in the saturated to unsaturated FAs ratios associated with decreases in the tissue's antioxidant defenses [12,13].

The PVAT redox state was also modified by the fructose overload, as shown by a) a decreased activity of key antioxidant enzymes such as SOD and GSH-Px, which account for the excessive production of superoxide anion and organic peroxides, respectively, b) a reduction in glutathione content, the major antioxidant of water-soluble cellular components, c) an increased GSH-Rd activity that likely reflects a compensatory mechanism to return oxidized glutathione to its reduced state, and d) a higher NO production, which may reflect the activation of the inducible isoform of NO synthase in response to the augmented oxidative stress. Altogether, these alterations decreased the cell capacity to cope with oxidative stress and are responsible for the recorded increase in biomarkers of oxidative damage, mainly lipid peroxidation products, estimated through the TBARS concentration in PVAT. As mentioned before, excessive chain breaking produced by reactive species is reflected in the altered saturated/unsaturated FAs ratio present in PVAT triglycerides. These changes resemble those reported in abdominal adipose tissue [12,13,15], thus suggesting that they are the consequence of a general rather than a single local gluco-oxidative stress and can partly explain the association between adipose tissue and vascular dysfunction.

The presence of increased oxidative stress in different tissues of IR and diabetic animals has also been reported, such as a NO synthase dysfunction in Goto-Kakizaki rats, which could be attenuated by exercise [46], and increased ROS production in aortas of streptozotcin-diabetic mice, that was reverted by aminoguanidine administration [47].

#### Vascular reactivity

It has been reported that IR states and type 2 diabetes are associated to the development of vascular complications, mostly related to hyperglycemia, dyslipidemia, protein glycation and enhanced oxidative stress [48]. All these alterations are present in FRD rats, and animals fed with such diet for 8 weeks presented endothelial dysfunction related to reduced NO synthesis and increased oxidative stress [49].

In our study, we tested whether the significant FRD-induced changes on PVAT lipid composition and redox status affected vessel contractility. For that purpose, we used both intact and PVAT-deprived aorta rings and two different stimuli: a) 5-HT, because it induces a PVAT-dependent relaxant response [24], and b) depolarization by high extracellular K^+^, because it does not provoke relaxation in a PVAT-dependent fashion. Results showed that removal of PVAT increased the force developed by aortic rings in response to 5-HT; however, such increase was significantly smaller in rings from FRD rats. This effect was no longer observed when the two contractions were performed in PVAT-deprived rings. On the other hand, no differences were recorded in rings from CD and FRD rats in the contractile response to high extracellular K^+^, neither using intact nor PVAT-deprived rings. The latter effect was not unexpected, since factors released by PVAT would produce vascular smooth muscle relaxation through the hyperpolarization achieved by the opening of K^+ ^channels [24,26,28], and K^+ ^channel activation does not hyperpolarize the cell when the extracellular K^+ ^concentration is high. Interestingly, there would be regional differences in the type of K^+ ^channels activated by PVAT relaxing factors: while in some vessels there are high-conductance Ca^2+^-activated K^+ ^channels (BK_Ca_) [26], in others ATP-sensitive K^+ ^channels (K_ATP_) [24] or delayed-rectifier K^+ ^channels (K_V_) [28] are the target. We cannot rule out the possibility that agonists produced by the PVAT in FRD rats modified the vascular smooth muscle cells-IP3 receptor properties, as it occurred in rat models of type 1 diabetes [50].

As already mentioned, there were no differences in the contractile forces developed by 5-HT or by high extracellular K^+ ^between CD and FRD rings without PVAT (Figure 1B). Consequently, the current results suggest that in FRD rats, at least in the period studied, the paracrine PVAT function rather than the smooth muscle function was affected. Comparable results regarding aortic rings without PVAT were reported by Verma et al. [51]: these authors found that administration of a diet supplemented with a fructose concentration higher than ours (66% fructose) for 3 weeks did not affect vascular reactivity to noradrenaline.

The effect of PVAT upon vascular function could be ascribed to many molecules produced by this tissue. ROS, namely superoxide anion [30] and H_2_O_2 _[31] are two of the vasoactive substances released by PVAT. Thus, the change in the redox state (SOD and others) produced by the FRD will surely affect their concentrations. However, since superoxide anion promotes vessel contraction while H_2_O_2 _induces its relaxation, the final outcome will depend on their relative PVAT production/release balance. Further experiments are required to define whether the changes observed in the vascular response to 5-HT are the consequence of the increased ROS production/action or just casually associated. In this regard, it would also be interesting to study whether FRD administration for a longer period accentuates the currently described PVAT alterations.

Despite the significant association between gluco-oxidative stress markers and relaxation changes recorded, some other factors released by PVAT, such as leptin [52], could also play a role as a signal in our model. In this regard, we have already reported that FRD-fed rats have high serum leptin levels and that their abdominal adipose tissue has a higher expression of its gene and releases larger amounts of leptin [13]. Considering that all the changes recorded in the parameters measured in PVAT are highly similar to those reported in abdominal adipose tissue, we could therefore assume that leptin's behaviour would follow the same pattern. Altogether, these results support the concept of the important role of the crosstalk between PVAT fat and vascular function [53,54].

Even when we found a great coincidence among gluco-oxidative stress markers, changes in PVAT composition and those measured in vascular properties, other factors such as hypertension - present in the FRD model [8] - could also participate in the underlying mechanism responsible for the latter. Anyhow, and as part of the complex net of interactions present in this animal model, the increased formation of ROS also contributes to fructose-induced hypertension.

### Final remarks and conclusions

The current results evidence that an increased intake of fructose induces multiple metabolic and endocrine systemic alterations which are concomitant with significant changes in PVAT fatty acyl composition and oxidative stress biomarkers. These changes were similar to those measured in abdominal adipose tissue in this rat model. While at this stage (3 weeks of treatment) the vascular smooth muscle response was still unaffected, alterations in the vascular relaxant properties dependent on the PVAT were already evident. These results should contribute to elucidate, at least partly, the strong association between adipose tissue and vascular dysfunction. They also suggest that at early stages of this rat model that resemble the human MS, alterations in the paracrine function of PVAT precede those of vascular smooth muscle. Finally, the FRD rat model represents a valuable experimental tool to study the effect of agents potentially able to prevent or attenuate the vascular pathology associated with the MS and type 2 diabetes. Further, high fructose diets certainly promote multiple undesirable metabolic and vascular disorders.

## Competing interests

The authors declare that they have no competing interests.

## Authors' contributions

AR and JJG conceived and designed the study, performed the statistical analyses and drafted the manuscript. AR, ARRP and LR carried out the vascular reactivity experiments. CAM performed the enzymatic and lipid peroxidation assays. ORR and MEG carried out fructosamine, lipid, triglyceride and lipid peroxidation determinations. All authors read and approved the final manuscript.
